# Functional Evidence for a Dual Route to Amygdala

**DOI:** 10.1016/j.cub.2011.11.056

**Published:** 2012-01-24

**Authors:** Marta I. Garrido, Gareth R. Barnes, Maneesh Sahani, Raymond J. Dolan

**Affiliations:** 1 Wellcome Trust Centre for Neuroimaging, University College London, London WC1N 3BG, UK; 2 Gatsby Computational Neuroscience Unit, University College London, London WC1E 3AN, UK

## Abstract

The amygdala plays a central role in evaluating the behavioral importance of sensory information. Anatomical subcortical pathways provide direct input to the amygdala from early sensory systems and may support an adaptively valuable rapid appraisal of salient information [1–3]. However, the functional significance of these subcortical inputs remains controversial [4]. We recorded magnetoencephalographic activity evoked by tones in the context of emotionally valent faces and tested two competing biologically motivated dynamic causal models [5, 6] against these data: the dual and cortical models. The dual model comprised two parallel (cortical and subcortical) routes to the amygdala, whereas the cortical model excluded the subcortical path. We found that neuronal responses elicited by salient information were better explained when a subcortical pathway was included. In keeping with its putative functional role of rapid stimulus appraisal, the subcortical pathway was most important early in stimulus processing. However, as often assumed, its action was not limited to the context of fear, pointing to a more widespread information processing role. Thus, our data supports the idea that an expedited evaluation of sensory input is best explained by an architecture that involves a subcortical path to the amygdala.

## Highlights

► Salient information processing engages cortical and subcortical routes to amygdala ► The subcortical pathway is important in early processing stages ► Expedited subcortical processing is not limited to fear related stimuli

## Results

Our goal was to assess the explanatory power of a fast subcortical route in salient information processing. We first investigated whether brain responses elicited by a salient context, such as unpredictable information under threat, were better modeled with or without a subcortical “low route.” We hypothesized that early evoked responses would be better explained by the dual-route model and predicted that a subcortical pathway would play a more significant role in early, rather than later, time epochs. The critical factor in such a model is rapidity of processing, and this mandates a methodology with adequate temporal resolution. Thus, we used computational modeling to compare models, with and without the subcortical pathway, and evaluated their predictions in terms of how well they explained evoked magnetoencephalographic (MEG) data. In addition, we asked whether the functional role of the subcortical pathway depends on stimulus predictability and emotional context. This provides an opportunity to address an unresolved and controversial question as to the degree to which subcortical processing promotes expeditious evaluation of biological significance in sensory information.

### Surprise-Evoked Fields Are Enhanced in a Fearful Context

**Figure 1:**
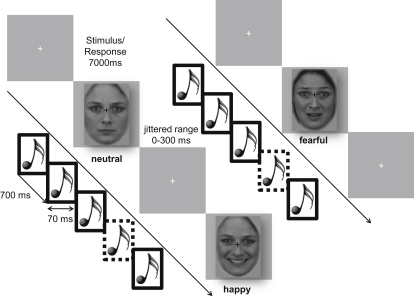
Experimental Design A passive auditory oddball paradigm was presented while participants performed a visual gender discrimination task. Standard (1,000 Hz) and deviant (1,100 Hz) sounds lasted for 70 ms and were played every 700 ms with 90% and 10% probability, respectively. Contextual emotional information was manipulated by 7 s long visual presentation of neutral, happy, and fearful faces.

**Figure 2:**
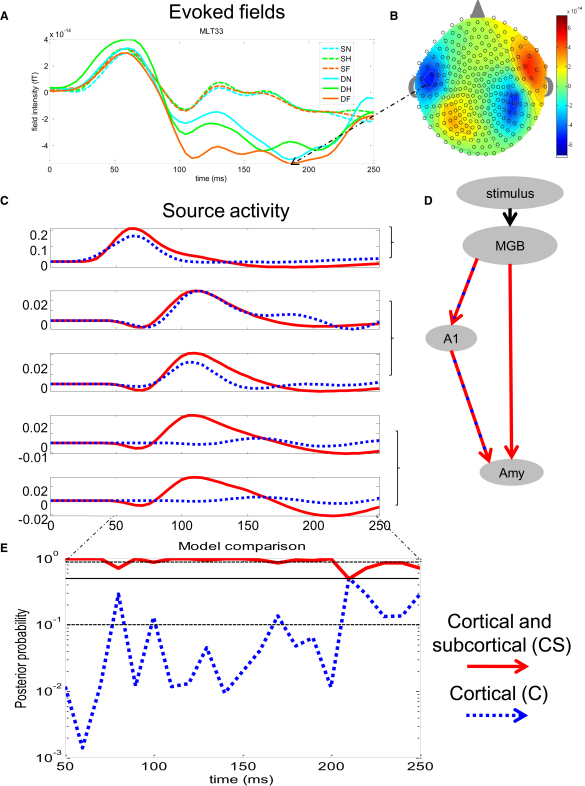
Cortical and Subcortical Pathways of Salient Information (A) Grand-mean data (n = 12) show enhanced responses to surprising compared to predictable auditory events. Responses to predictable sounds were similar across contextual manipulation of facial expressions. Surprised evoked fields increased with the emotional salience of facial expressions and were most deflected in the context of fearful faces. (B) Scalp topography for surprise-evoked fields in a fearful context peaking at 185 ms showed a bilateral dipolar pattern over the temporal cortex. (C) Source activity predicted by the dual-route (CS) (in red) and the cortical (C) (in blue) models at all network nodes shows enhanced early amygdala activity for model CS as compared to (C), whereas activity in auditory cortex remains similar. (D) Graphical description of the models. Model CS includes both cortical and subcortical pathways that convey information from the auditory thalamus (MGB) indirectly, (through A1), or directly to the amygdala. Model (C) includes the cortical pathway only, precluding the subcortical pathway to amygdala. (E) Bayesian model comparison reveals that the dual-route model explains the group data overall better than the cortical model alone, especially in early temporal windows. Solid black line corresponds to 50% probability, and the dotted black lines correspond to 90% and 10% probabilities. See also Figures S1 and S2.

We presented participants with a sequence of predictable and surprising pure tone sounds. Subjects simultaneously performed a gender discrimination task on visually presented faces with neutral, happy, or fearful expressions (Figure 1). Responses to predictable, or standard, sounds were similar in all three contexts. However, the strength of the fields evoked by oddballs, or surprising events, increased with the emotional salience of facial expressions. This gradient was particularly evident in the period of 100–150 ms poststimulus, with the largest effect being evident in the context of fearful faces, consistent with previous studies [7] (Figure 2A).

### Enhanced Early Amygdala Activity with a Subcortical Pathway

We estimated that activity at each source included in two competing dynamic causal models (DCMs) [5] for oddballs under fear (Figure 2C and 2D). The cortical model (C) included a cortical pathway only, which tests a hypothesis that information about auditory objects reaches the amygdala after being processed by the auditory thalamus (MGB) and primary auditory cortex (A1). On the other hand, a dual-route, or cortical and subcortical model (CS), included a cortical and subcortical pathway, expressing a hypothesis that information reaches the amygdala both directly through a thalamic projection and indirectly through a cortical route (Figure 2D).

Activity in A1 as estimated by both models was similar. Crucially, we found that the dual-route model could recover early amygdala activity (peaking at ∼50 ms and ∼100 ms). Conversely, the absence of the subcortical pathway linking MGB to AMY caused early (<100 ms) amygdala activity to disappear. The cortical model could only recover late amygdala activity (peaking at about 150 ms) (Figure 2C). This dissociation supports the role of a subcortical pathway in conveying rapid information to the amygdala.

### Time-Specific Role of the Subcortical Pathway

Neuroanatomical tracings in the rat demonstrate the existence of two parallel processing pathways involving a thalamo-cortico-amygdala and a direct thalamo-amygdala pathway [8]. There is also evidence that auditory inputs can access the basolateral amygdala from both the auditory thalamus and the cortex [9–11]. Crucially, direct subcortical connections between the auditory thalamus and the amygdala are alone sufficient for some forms of fear conditioning [12–14]. On this basis it is argued that a subcortical pathway plays an important role in adaptive behavior. Indeed, the ability to rapidly process behaviorally relevant information represents a biological advantage in a potentially dangerous environment. Hence, a fast route that bypasses cortical processing is central to the dual-route hypothesis [2]. Motivated by this and the source analysis described above (Figure 2C), we asked whether the relevance of the subcortical pathway was dependent on time. We hypothesized that the functional role of the subcortical pathway is crucial at early processing stages and predicted that early data should be better explained by a model with, than without, a subcortical pathway. To test this, we considered an increasing time window of data to model every participant's responses with the dual-route (CS) and the cortical model alone (C). This time window was initially set to [0–50] ms and gradually increased in steps of 10 ms to cover a total time window of [0–250] ms.

Bayesian model comparison [15] revealed that the dual-route model, CS, explains the group data better than the cortical model alone, C, especially at early temporal windows. The median probability for CS in early temporal windows (<200 ms) was 98% and in late temporal windows (>200 ms) was only 70%. In later time periods, either model C or CS won but with a probability barely above chance (Figure 2E). Thus, these results demonstrate that the subcortical pathway is crucial in explaining data observed at earlier time periods, shortly after stimulus onset, whereas for later periods the advantage of a dual over a cortical model is not as clear. Indeed, in these wider time windows, no model performs significantly better than the other.

### The Role of the Subcortical Pathway in General Information Processing

**Figure 3:**
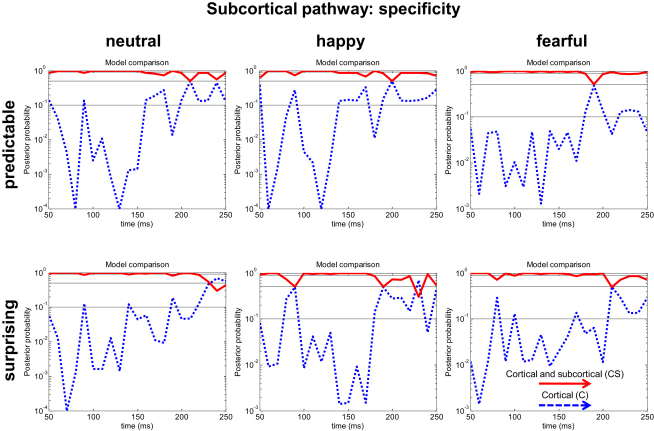
Subcortical Pathway Specificity Bayesian model comparison revealed that the dual-route model (in red) explains the group data overall better than the cortical model (in blue) alone, across all conditions (predictable and surprising under the different emotional contexts—neutral, happy, and fearful), especially in early temporal windows.

We further investigated whether the superiority of the dual-route model was specific to the maximally salient condition (unpredictable sounds in the context of a fearful face) or common to all conditions. To our surprise, we found that the time-dependent relevance of the subcortical pathway was general to all sensory processing. The median probability for CS in the predictable conditions was 87%, 87%, and 95% in the surprising and 95%, 95%, and 96% for the neutral, happy, and fearful conditions, respectively (see Figure 3).

Thus, these results address the temporal and anatomical predictions of a dual-route and demonstrate that such a model outperforms a cortical model, being especially important in explaining activity during early temporal windows. Moreover, the relevance of the subcortical pathway seems to be a general phenomenon, regardless of the specific emotional context and predictability, rather than being specific to the context of fear.

## Discussion

By providing an explicit statistical test for the necessity of a subcortical pathway, we show that processing of salient events is consistent with the idea of a dual-route to the amygdala. With Bayesian model comparison, we show that a model incorporating a subcortical pathway better explains group and individual data than a model with a cortical pathway alone. This subcortical pathway was particularly important in earlier processing periods, in line with its putative adaptive role (Figure 2E). Moreover, we show that the dual-route model could reliably recover early amygdala activity (Figure 2C). In addition, we found that the subcortical pathway plays a fundamental role in conveying information to the amygdala, regardless of stimulus predictability and irrespective of the emotional context in which they appear (Figure 3). The findings are in keeping with the view that a “low” route promotes an expeditious evaluation of biological significance in sensory information.

To test the robustness of these results, we performed a number of validity checks. First, we compared the accuracy of the models with and without the subcortical pathway and found that indeed the dual model explained the MEG channel data better than the cortical model alone (r_CS_ = 0.98 versus r_C_ = 0.93, see Figure S1 available online). In order to test for the specificity of the MEG data to amygdala activity, we performed an additional analysis where the amygdala was replaced by other plausible regions (Figure S2). These regions were bilateral hippocampus (HIPP model), two extra bilateral sources around A1 (A1+), bilateral inferior colliculus (IC), and bilateral superior temporal gyrus (STG). We then considered a similar model to the latter, where forward connections were removed from A1 to STG (STG_nf). We found that the AMY model was the best among all models, outperforming the second most likely model, STG_nf, with very strong evidence [16]. This comparison also demonstrates that amygdalar and hippocampal sources can be discriminated, thereby adding to the confidence in our inference that these reconstructed signals do indeed emanate from the amygdala and not from a neighboring deep-brain source.

As a final check, we performed simulations that assessed the relative sensitivity of our MEG system to these deeper structures. The sensitivities of the MEG system to the amygdala, hippocampus, and STG, relative to the auditory cortex, were 92% ± 3%, 62% ± 2%, and 182% ± 7%, respectively. This demonstrates that we do not lose much sensitivity in the amygdala when compared to A1. In fact, MEG sensitivity to A1 is already relatively small when compared to the visual or somatosensory cortex [17]. We also note that recent MEG studies [7, 18–21] report being able to reconstruct activity in the amygdala and hippocampus, as well as in thalamic [22] and brainstem structures [23].

Our results support the dual-route hypothesis [2, 8, 12]. Evidence for a subcortical route includes, for example, data showing enhanced thalamus-amygdala coupling during processing of masked fearful stimuli [24], and enhanced amygdala activity to unseen fear in a patient with blindsight [25], presumably generated through subcortical thalamic-amygdala projections. We also note that a patient with complete cortical blindness exhibited startle reflexes potentiated in the presence of a conditioned visual stimulus and not prior to conditioning [26]. In line with this result, it has been shown that a cortically blind patient could behaviorally discriminate emotional faces above chance, and emotional discrimination was correlated with right amygdala activity [27]. Interestingly, although the effect was higher in a fearful context, successful emotional discrimination and amygdala activation were present for all emotional expressions, regardless of their specific emotional content. This points to a more general (rather than fear-specific) functional role for the subcortical pathway to the amygdala, consistent with our demonstration that the dual-route model best explains early neuronal responses evoked by either predictable or unpredictable stimuli presented in any emotional context (fearful, happy, or neutral—see Figure 3). In this sense, our findings converge on the idea that expedited processing is not specific to affective information [4]. M/EEG [7, 28, 29] and monkey electrophysiology studies [30] fail to demonstrate evidence for differences in the timing of initial stimulus responses to salient stimuli (even if reliable amplitude differences are reported at 100–200 ms). Again, this suggests that the role of the subcortical pathway might not be specific to emotionally salient stimuli, but rather, general to sensory information.

Whereas previous work does not make any strong claim that the subcortical pathway only applies to fear, most of this work has tended to use fear paradigms, as in the seminal work of LeDoux et al. [9]. Therefore, the belief that the dual model should be specific for fear might simply result from the paradigms typically used to explore it. However, it remains unclear what might be driving the larger response to a deviant in the fearful context observed in the sensor data. We investigated possible effects on the coupling among the network regions, and also on the estimated source activity, but found no clear evidence. Previous functional magnetic resonance imaging (fMRI) studies have found significant differences (with visual stimuli) between fearful and neutral conditions; however, the slow blood-oxygen-level dependent (BOLD) signal is likely to reflect late amygdala responses [6, 20, 31] when recurrent activity is expected to occur [6, 32]. On the contrary, MEG has sensitivity to early (automatic) amygdala activity and fMRI and MEG measurement differences might be core to the apparent conflicting results [3, 20, 33, 34] (see also [32] for a critical review). This remains an interesting issue for further investigation. We should also point out that although our results are consistent with the related literature on salient visual processing, our data have no bearing on whether brain responses evoked by complex visual stimuli, like faces, are processed by a “low” visual route.

### Conclusion

In summary, using model comparison we show that a dual-route model best explained neuronal responses to sensory stimuli. We show that a subcortical route is both time-dependent and crucial in explaining earlier processing stages. In addition, this subcortical pathway causes short-latency amygdala activation, which would otherwise be delayed, in keeping with an expedited processing of relevant information and rapid engagement of an appropriate behavioral response. On this basis, our results provide novel insights into the mechanistic and functional role of a putative “low” route.

## Experimental Procedures

### Participants

We recorded whole-head MEG data from 12 healthy naive participants. The experimental procedures were approved by the University College London Hospitals Ethics Committee.

### Experimental Design

The paradigm was adapted from a previous study [35] (see Figure 1). During the incidental gender discrimination task (with neutral, happy, and fearful faces), participants were simultaneously presented with an auditory frequency oddball paradigm.

### Model Specification and Statistical Inference

Here, we tested two dynamic causal models (DCMs) [5, 36] that map onto two candidate models or hypotheses: the dual-route model and the cortical model. The dual-route model included both cortical and subcortical pathways, which convey information from the auditory thalamus (MGB) directly or indirectly (through A1) to the amygdala. The cortical model included the cortical pathway alone, hence excluding subcortical connections to the amygdala (Figure 2D). We used an increasing time window approach (as described in [6], Figure 2E). This approach attempted at addressing whether the usefulness of the subcortical pathway was time specific. Statistical inference on models was implemented using a Bayesian random effects approach [15]. For details on experimental procedures see Supplemental Information.

## Supplemental Information

Document S1. Figures S1 and S2 and Supplemental Experimental Procedures
