# Supplementary material for: Functional Evidence for a Dual Route to Amygdala
Source: Curr Biol. 2012 Jan 24;22(2-2):129–34. doi: 10.1016/j.cub.2011.11.056 (PMC3267035; doi:10.1016/j.cub.2011.11.056)
Supplement: Document S1. Figures S1 and S2 and Supplemental Experimental Procedures [file mmc1.pdf]

Current Biology, Volume *22*

Supplemental Information

Functional Evidence for a Dual Route to Amygdala

Marta I. Garrido, Gareth R. Barnes, Maneesh Sahani, and Raymond J. Dolan

Supplemental Inventory

1. Supplemental Figures and Tables

Figure S1, related to Figure 2

Figure S2, related to Figure 2

2. Supplemental Experimental Procedures

3. Supplemental References

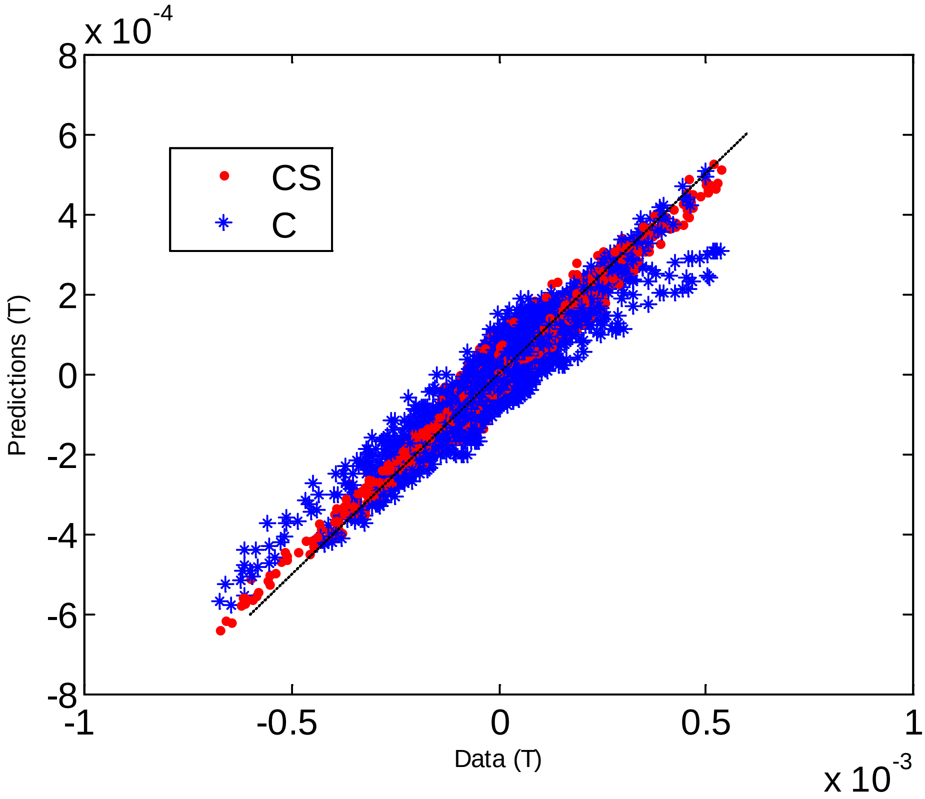

Figure S1. Correlation of Data Features and Model Predictions, Related to Figure 2
The dual-model explains the grand mean MEG channel data better than the cortical model alone (r_{CS}=0.98 vs. r_C=0.93), which illustrates the improvement in accuracy implicit in the free energy calculation.

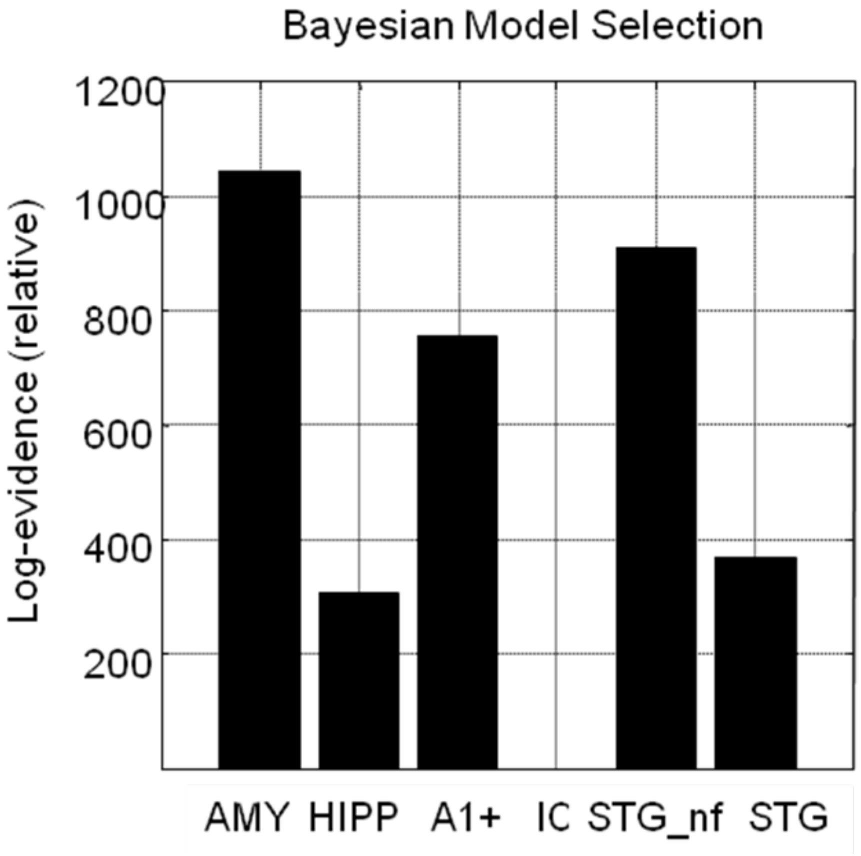

Figure S2. Amygdala Specificity, Related to Figure 2

Bayesian model comparison of the dual-model based on the amygdala location (AMY) clearly outperformed all other models tested. The amygdala locations were replaced by the hippocampus (HIPP), extra bilateral sources around A1 (A1+), bilateral inferior colliculus (IC), bilateral superior temporal gyrus (STG); and finally, a similar model with forward connections removed from A1 to STG (STG_nf). AMY model was the best amongst all models, outperforming STG_nf (second most likely model) with very strong evidence (log Bayes factor>5 [1]).

Supplemental Experimental Procedures

Participants

We recorded MEG data from twelve healthy naïve participants (3 males, 9 females, age range 24–35 years, and mean age 31.4 years). All participants reported normal hearing and normal or corrected to normal vision. Informed consent was obtained from each subject, after full explanation of the experiment, according to the procedures approved by the University College London Hospitals Ethics Committee. Participants were monetarily compensated for their time.

Experimental Design

The paradigm was adapted from a previous study [2] and involved a 2 (sounds: standards, deviants) x 3 (contextual faces: neutral, happy, fearful) design. Participants sat comfortably in front of a computer screen in a dimly-illuminated magnetically shielded room, while they performed a gender discrimination task on visually presented faces, by means of a button press. Photos of male and female faces were of equal number. Faces were presented in a randomised sequence, each for a sustained period of 7s. There was a jittered period of 0-300ms between presentations of each face (see Figure 1). A total of 54 faces were selected from [3], 18 per facial expression (9 females and 9 males). Hair and ears were removed from the greyscale photos to make the task more difficult.

During the incidental judgement task, participants were simultaneously presented with an auditory frequency oddball paradigm. The oddball paradigm is characterised by a regular sequence of pure tones that occasionally varied in their frequency [4]. The most prevalent, or standard sounds were played at 1000 Hz with 90% probability and the rare, or deviant sounds were played at 1100 Hz with 10% probability. Sounds were played every 700ms and lasted 70 ms. Hence, the emotional expression of a face provided a contextual background in which both standard and deviant sounds were heard. There were a total of 99 oddball and 990 standard trials per contextual condition for most participants, the exception being 2 participants who performed only 2 out of 3 consecutive experimental sessions. Prior to the actual experiment, all subjects performed a 3 minute practice session, in which they became familiar with the stimuli and the task. The photos used in the practice session were not included in the actual experiment. Participants were instructed to ignore the sounds and the emotional expressions of the observed faces.

The stimulus control and task software were written in MATLAB, using the Cogent 2000 toolbox (http://www.vislab.ucl.ac.uk/cogent.php).

MEG Recordings and Preprocessing

Measurements were acquired with a CTF 275-channel whole-head MEG system, with 274 functioning second-order axial gradiometers arranged in a helmet shaped array. Data were collected at a sampling rate of 600 Hz. Subsequently, data were off-line down-sampled to 200 Hz, bandpass filtered from 0.5 to 30 Hz, and baseline corrected with reference to [-100–0] ms. Averages were time-locked to sound onsets. Three energised electrical coils were attached to the fiducials (nasion, and left and right preauricular), in order to continuously monitor the position of each participants head with respect to the MEG sensors.

Auditory stimuli were binaurally presented at a comfortable loudness level, through a flexible tubing connected to piezo electric transducers positioned approximately 1m below the sensor array.

Model Specification and Statistical Inference

Dynamic causal modelling (DCM) is a hypothesis driven approach to the analysis of brain connectivity. Originally developed for functional magnetic resonance imaging (fMRI) data [5], it has subsequently been extended for electrophysiological data such as those observed with EEG, MEG and local field potentials (LFP) [6-10]. As opposed to data-driven approaches useful for network discovery [11-13], DCM has a powerful use in testing competing hypotheses or models that generate data in a specific context [14-22]. This class of questions, ill-posed by nature, can be powerfully addressed in a Bayesian framework that allows for model selection amongst alternatives, and inference about the directionality of specific connections as well as their parameterisation.

Here, we tested two DCMs that map onto two candidate models or hypotheses: the dual-route model (CS) and the cortical model (C). These hypotheses attempt to explain how salient information is processed in the brain, and more specifically which pathways might convey this sort of information to the amygdala [23-25]. Therefore, we initially focused our analysis on the ERF evoked by surprising (oddballs) trials in the context of fearful faces, and then reproduced the same analyses for the remaining five conditions. Both models included four areas modelled as an equivalent current dipole (ECD) a priori fixed locations placed over the left (-30,-2,-13) and right (23,-2,-23) amygdala (AMY) [26], and the left (-42,-22,7) and right (46,-14,8) primary auditory cortex (A1). In addition, we included a hidden source to emulate activity in the medial geniculate body (MGB), known to respond differently to outliers [27], which was also modelled as an ECD but with no contribution to scalp activity. These regions were connected with forward, or bottom-up, connections according to the rules described in [28, 29]. We reduced the data to their eight principal components through singular value decomposition, and used one discrete cosine transform component to remove slow drift. We posited no constraints on the symmetry of dipolar orientation or on within-area connectivity. The dual-route model (CS) included both cortical and subcortical pathways, which convey information from the auditory thalamus (MGB) directly or indirectly (through A1) to the amygdala. The C model included the cortical pathway alone, hence, precluding subcortical connections to the amygdala (Figure 2D). Given that we were interested in testing the idea of a dual-route model with a subcortical pathway allowing for rapid processing of significant information, we then compared these models against each other as a function of time. For that we used an increasing time window approach (described in [14]) to model data observed in [0-50] ms, and thereafter in steps of 10 ms up to [0-250] ms (Figure 2E). This approach attempted at addressing whether the usefulness of the subcortical pathway was time specific.

Statistical inference on models was implemented using a random effects approach [30, 31] to compute a group Bayes factor from each subject and each models negative free energy. As opposed to fixed effects [8], random effects analysis models outlier effects, a providing less biased estimates of group Bayes factors.

All the analysis was done with SPM and in-house MATLAB scripts.

Validation Checks

To further test the robustness of our results we performed a number of validity checks. Firstly, we compared the accuracy of the models with and without the subcortical pathway and found that indeed the dual-model explained the MEG channel data better than the cortical model alone (r_{CS}=0.98 vs. r_{C}=0.93, see Figure S1). As the dual model contains more free parameters this was not unexpected; but it illustrates the improvement in accuracy implicit in the free energy calculation. In order to verify that an improvement in observed free energy

could not be simply due to more available parameters to fit, and to test for the specificity of the MEG data to amygdala activity, we performed an additional identical analysis where the amygdala was replaced by other plausible regions (Figure S2). These models were constrained by biologically motivated priors on spatial source locations and dynamics and assessed how well these models explained the grand mean data observed in the first 130 ms after stimulus onset. First, we replaced the amygdalae by left (-27,-30,-3) and right (27,-30,-3) hippocampus (HIPP model). We then replaced them by two extra bilateral sources around A1 (A1+ model, (-45,-25,10) and (49,-17,11)). In a different model, the amygdalae were replaced by bilateral inferior colliculus (IC model, (-4,-34,-12) and (6,-35,-12), [32]). Two further models included the bilateral superior temporal gyrus (STG) instead of the amygdalae, one with and one without (STG_nf) forward connections from A1 to STG. We found that the AMY model was the best amongst all the alternatives, outperforming the second most likely model, STG_nf, with very strong evidence (log Bayes factor >5 [1]). This analysis shows that the amygdala model explains measured MEG data much better than a model in which the amygdala was replaced by alternative deep sources (and indeed better than models with additional auditory or inferior colliculus sources). Furthermore, the analysis demonstrates that amygdalar and hippocampal sources can be discriminated, thereby adding to the confidence in our inference that these reconstructed signals do indeed emanate from the amygdala, and not from a neighbouring deep-brain structure.

As a final check, we performed a simulation study in which we assessed the relative sensitivity of our MEG system to these deeper structures (based on geometrical information alone and ignoring cortical architecture). We computed the relative lead field magnitudes within the amygdala, the hippocampus, and the STG as compared to A1. The sensitivities of the MEG system to voxels within the amygdala, hippocampus and STG relative to the auditory cortex were 92±3%, 62±2%, and 182±7% respectively. This demonstrates that we do not lose much sensitivity in the amygdala when compared to A1. In fact, MEG sensitivity to A1 is already relatively small when compared to the visual or somatosensory cortex [33]. We also note recent MEG studies [26, 34-36] report being able to reconstruct activity in amygdala and hippocampus, as well as reports that thalamic [37] and brainstem structures [38] can also be reliably identified. Here, we also gain a degree of immunity to noise by using a model that incorporates constraints on the cortical dynamics.

Supplemental References

1. Penny, W.D., Stephan, K.E., Mechelli, A., and Friston, K.J. (2004). Comparing dynamic causal models. Neuroimage *22*, 1157-1172.
2. Surakka, V., Tenhunen-Eskelinen, M., Hietanen, J.K., and Sams, M. (1998). Modulation of human auditory information processing by emotional visual stimuli. Brain Res Cogn Brain Res *7*, 159-163.
3. Ekman, P., and Friesen, W. (1975). Pictures of facial affect. (Palo Alto, California: Consulting Psychologist Press).
4. Naatanen, R., Teder, W., Alho, K., and Lavikainen, J. (1992). Auditory attention and selective input modulation: a topographical ERP study. Neuroreport *3*, 493-496.
5. Friston, K.J., Harrison, L., and Penny, W. (2003). Dynamic causal modelling. Neuroimage *19*, 1273-1302.
6. David, O., Kilner, J.M., and Friston, K.J. (2006). Mechanisms of evoked and induced responses in MEG/EEG. Neuroimage *31*, 1580-1591.
7. Kiebel, S.J., David, O., and Friston, K.J. (2006). Dynamic causal modelling of evoked responses in EEG/MEG with lead field parameterization. Neuroimage *30*, 1273-1284.
8. Garrido, M.I., Kilner, J.M., Kiebel, S.J., Stephan, K.E., and Friston, K.J. (2007). Dynamic causal modelling of evoked potentials: a reproducibility study. Neuroimage *36*, 571-580.
9. Chen, C.C., Kiebel, S.J., and Friston, K.J. (2008). Dynamic causal modelling of induced responses. Neuroimage *41*, 1293-1312.
10. Moran, R.J., Stephan, K.E., Seidenbecher, T., Pape, H.C., Dolan, R.J., and Friston, K.J. (2009). Dynamic causal models of steady-state responses. Neuroimage *44*, 796-811.
11. Roebroeck, A., Formisano, E., and Goebel, R. (2009). The identification of interacting networks in the brain using fMRI: Model selection, causality and deconvolution. Neuroimage.
12. Ramsey, J.D., Hanson, S.J., Hanson, C., Halchenko, Y.O., Poldrack, R.A., and Glymour, C. (2010). Six problems for causal inference from fMRI. Neuroimage *49*, 1545-1558.
13. Ramsey, J.D., Hanson, S.J., and Glymour, C. (2011). Multi-subject search correctly identifies causal connections and most causal directions in the DCM models of the Smith et al. simulation study. Neuroimage.
14. Garrido, M.I., Kilner, J.M., Kiebel, S.J., and Friston, K.J. (2007). Evoked brain responses are generated by feedback loops. Proc Natl Acad Sci U S A *104*, 20961-20966.
15. Garrido, M.I., Friston, K.J., Kiebel, S.J., Stephan, K.E., Baldeweg, T., and Kilner, J.M. (2008). The functional anatomy of the MMN: a DCM study of the roving paradigm. Neuroimage *42*, 936-944.
16. Garrido, M.I., Kilner, J.M., Kiebel, S.J., and Friston, K.J. (2009). Dynamic causal modeling of the response to frequency deviants. J Neurophysiol *101*, 2620-2631.
17. Garrido, M.I., Kilner, J.M., Kiebel, S.J., Stephan, K.E., Baldeweg, T., and Friston, K.J. (2009). Repetition suppression and plasticity in the human brain. Neuroimage *48*, 269-279.
18. Schofield, T.M., Iverson, P., Kiebel, S.J., Stephan, K.E., Kilner, J.M., Friston, K.J., Crinion, J.T., Price, C.J., and Leff, A.P. (2009). Changing meaning causes coupling changes within higher levels of the cortical hierarchy. Proc Natl Acad Sci U S A *106*, 11765-11770.
19. Dima, D., Dietrich, D.E., Dillo, W., and Emrich, H.M. (2010). Impaired top-down processes in schizophrenia: a DCM study of ERPs. Neuroimage *52*, 824-832.
20. David, O., Guillemain, I., Saillet, S., Reyt, S., Deransart, C., Segebarth, C., and Depaulis, A. (2008). Identifying neural drivers with functional MRI: an electrophysiological validation. PLoS Biol *6*, 2683-2697.

21. Boly, M., Garrido, M.I., Gosseries, O., Bruno, M.A., Boveroux, P., Schnakers, C., Massimini, M., Litvak, V., Laureys, S., and Friston, K. (2011). Preserved feedforward but impaired top-down processes in the vegetative state. Science *332*, 858-862.
22. Campo, P., Garrido, M.I., Moran, R.J., Maestu, F., Garcia-Morales, I., Gil-Nagel, A., Del Pozo, F., Dolan, R.J., and Friston, K.J. (2011). Remote Effects of Hippocampal Sclerosis on Effective Connectivity during Working Memory Encoding: A Case of Connectional Diaschisis? Cereb Cortex.
23. LeDoux, J.E. (2000). Emotion circuits in the brain. Annu Rev Neurosci *23*, 155-184.
24. Vuilleumier, P., Armony, J.L., Driver, J., and Dolan, R.J. (2003). Distinct spatial frequency sensitivities for processing faces and emotional expressions. Nat Neurosci *6*, 624-631.
25. Pessoa, L., and Adolphs, R. (2010). Emotion processing and the amygdala: from a 'low road' to 'many roads' of evaluating biological significance. Nat Rev Neurosci *11*, 773-783.
26. Cornwell, B.R., Baas, J.M., Johnson, L., Holroyd, T., Carver, F.W., Lissek, S., and Grillon, C. (2007). Neural responses to auditory stimulus deviance under threat of electric shock revealed by spatially-filtered magnetoencephalography. Neuroimage *37*, 282-289.
27. Anderson, L.A., Christianson, G.B., and Linden, J.F. (2009). Stimulus-specific adaptation occurs in the auditory thalamus. J Neurosci *29*, 7359-7363.
28. Felleman, D.J., and Van Essen, D.C. (1991). Distributed hierarchical processing in the primate cerebral cortex. Cereb Cortex *1*, 1-47.
29. David, O., Harrison, L., and Friston, K.J. (2005). Modelling event-related responses in the brain. Neuroimage *25*, 756-770.
30. Stephan, K.E., Penny, W.D., Daunizeau, J., Moran, R.J., and Friston, K.J. (2009). Bayesian model selection for group studies. Neuroimage *46*, 1004-1017.
31. Penny, W.D., Stephan, K.E., Daunizeau, J., Rosa, M.J., Friston, K.J., Schofield, T.M., and Leff, A.P. (2010). Comparing families of dynamic causal models. PLoS Comput Biol *6*, e1000709.
32. Budd, T.W., Hall, D.A., Goncalves, M.S., Akeroyd, M.A., Foster, J.R., Palmer, A.R., Head, K., and Summerfield, A.Q. (2003). Binaural specialisation in human auditory cortex: an fMRI investigation of interaural correlation sensitivity. Neuroimage *20*, 1783-1794.
33. Hillebrand, A., and Barnes, G.R. (2002). A quantitative assessment of the sensitivity of whole-head MEG to activity in the adult human cortex. Neuroimage *16*, 638-650.
34. Cornwell, B.R., Carver, F.W., Coppola, R., Johnson, L., Alvarez, R., and Grillon, C. (2008). Evoked amygdala responses to negative faces revealed by adaptive MEG beamformers. Brain Res *1244*, 103-112.
35. Riggs, L., Moses, S.N., Bardouille, T., Herdman, A.T., Ross, B., and Ryan, J.D. (2009). A complementary analytic approach to examining medial temporal lobe sources using magnetoencephalography. Neuroimage *45*, 627-642.
36. Luo, Q., Holroyd, T., Majestic, C., Cheng, X., Schechter, J., and Blair, R.J. (2010). Emotional automaticity is a matter of timing. J Neurosci *30*, 5825-5829.
37. Timmermann, L., Gross, J., Dirks, M., Volkmann, J., Freund, H.J., and Schnitzler, A. (2003). The cerebral oscillatory network of parkinsonian resting tremor. Brain *126*, 199-212.
38. Parkkonen, L., Fujiki, N., and Makela, J.P. (2009). Sources of auditory brainstem responses revisited: contribution by magnetoencephalography. Hum Brain Mapp *30*, 1772-1782.
